# A qualitative exploration of the barriers to and facilitators of clozapine monitoring in a secure psychiatric setting

**DOI:** 10.1192/bjb.2020.100

**Published:** 2021-06

**Authors:** Sarah Blagden, Jane Beenstock, Natalie Auld, Steve Noblett, Mark Limmer

**Affiliations:** 1 Lancashire and South Cumbria NHS Foundation Trust, UK; 2 Health Education North West, UK; 3 Lancaster University, UK

**Keywords:** Forensic mental health services, clozapine, antipsychotics, qualitative research

## Abstract

**Aims and method:**

To explore the beliefs and understanding of staff and patients at a secure mental health unit regarding clozapine monitoring, and to identify barriers to and facilitators of monitoring. Qualitative semi-structured interviews and focus groups were conducted with 17 staff members and six patients.

**Results:**

Six key themes were identified. The key facilitator of effective monitoring was the motivation of staff to help patients to become independent and facilitate recovery. An important barrier was a lack of clarity around the roles of different staff groups in monitoring. Staff and patients widely supported the establishment of an in-patient clozapine clinic and perceived that it would prepare patients for discharge.

**Clinical implications:**

An in-patient clozapine clinic is a robust mechanism for clozapine monitoring in secure settings. The barriers and facilitators identified here could be applied to other secure units to guide their systems of clozapine monitoring.

Clozapine is the only antipsychotic medication with established efficacy in adults with treatment-resistant schizophrenia and is an important treatment option in forensic psychiatric settings.^1^ However, it must be used with caution owing to its considerable side-effect profile.^1^ Most prominently, this includes a risk of neutropenia and fatal agranulocytosis, cardiac complications and bowel obstruction.^1^ Development of the metabolic syndrome, consisting of obesity, insulin resistance (often resulting in type 2 diabetes) and lipid derangements, is common.^1^ Consequently, alongside psychiatric monitoring, close monitoring of several physical health parameters is required for patients, as stipulated in the British National Formulary and by manufacturers (Table 1).

**Table 1:** Minimum physical health monitoring required for patients taking clozapine (source: BNF, Lancashire and South Cumbria NHS Foundation Trust^1^)

Monitoring for neutropenia	Weeks 1–18			Weeks 19–52		Ongoing monitoring
Full blood count	Weekly^a^	Every 2 weeks^a^		Every 4 weeks^a^
Other physical health parameters	Baseline	1 month	3 months	6 months	9 months	12 months/annually
Weight, BMI, waist circumference^b^	✓		✓	✓	✓	✓
Pulse and blood pressure^c^	✓					✓
HbA1C or fasting glucose	✓	✓		✓		✓
Prolactin	✓					✓
Lipids	✓		✓	✓	✓	✓
ECG^d^	✓					
Liver and renal function	Where there are concerns					

a. More frequent monitoring will be required if abnormal results are obtained.

b. Weight should be measured regularly during the first 3 months of clozapine treatment.

c. Blood pressure and pulse must be checked regularly during titration of clozapine.

d. If there are clear cardiac risk factors or an established cardiac comorbidity, troponin and C-reactive protein (CRP) should also be checked at baseline prior to initiation.

Despite being an important line of treatment, it is consistently reported in the literature that challenges remain around the use of clozapine. Previous audits have demonstrated incomplete adherence to physical health monitoring, particularly during the first year of monitoring when the risk of side-effects is greatest.^2–5^ Even where abnormalities are identified, this often does not translate into results being communicated or acted upon.^2,5,6^ A previous evaluation of shared-care clozapine monitoring found that implementing a different model of monitoring could feel process-driven and generate anxiety for staff, with a recommendation to identify facilitators and barriers to ensure that change is successful and sustained.^7^ Therefore, in this study, we aimed to explore the beliefs and understanding of staff and patients at a secure mental health unit regarding clozapine monitoring, and to use this information to identify barriers to and facilitators of monitoring.

## Methods

### Setting

The study setting was a secure mental health unit in north-west England. At the time of the study, approximately 30% of all patients were prescribed clozapine, with an average age of 36 years. This project was nested within a larger service evaluation of clozapine monitoring at the unit, which resulted in a clozapine clinic being recommended.

### Data collection

#### Staff

Semi-structured interviews and focus groups were conducted with 17 staff members. Purposive sampling was conducted in order to represent the different staff groups involved in clozapine monitoring.

First, all junior doctors based at the unit were invited by S.B. to participate in a focus group; all five agreed to participate. The physical health team, encompassing two general nurses and two healthcare support workers, also agreed to participate in a focus group. With regard to mental health ward staff, S.B. attended a selection of in-patient wards and opportunistically asked mental health staff (nurses and support workers) to take part in semi-structured interviews, which were conducted in a private room at the time of recruitment. Eight interviews were conducted. Interviews instead of focus groups were used for ward staff owing to the practical difficulties of multiple staff being simultaneously removed from clinical duties.

#### Patients

Semi-structured interviews were conducted with six patients. Again, purposive recruitment was performed to recruit patients from medium-secure, low-secure and step-down wards. Patients were approached by a mental health nurse, who introduced the project to the patients and accompanied S.B. during interviews. Only those patients that ward staff deemed clinically stable and able to provide informed consent were approached.

In all cases, the project's purpose and voluntary nature were explained, and verbal consent was obtained. As the primary purpose of the project was service evaluation, written consent was not deemed to be required when planning data collection with senior colleagues at the unit. Verbal consent was witnessed and formally recorded. It was required that participants spoke English and could provide verbal consent. Topic guides were used for interviews and focus groups and encompassed clozapine monitoring in general, with a possible clinic discussed at the end (see Appendices 1 and 2). The length of interviews ranged from 5 to 20 min, and focus groups lasted approximately 30 min.

### Epistemology

The research was underpinned by an interpretivist approach, which recognises the subjective nature of knowledge and the need to understand situations from the perspective of those involved.^8,9^

### Analysis

The focus group with doctors was audio-recorded and transcribed verbatim. All other data collection took place in clinical areas where it was not permitted to use audio-recording devices. Therefore, extensive field notes were made, and several quotes from each interviewee were transcribed verbatim to ensure data capture. Subsequently, based on field notes, quotes and transcripts, thematic analysis was utilised in the format described by Braun and Clarke.^8^ Thematic analysis is based on finding and interpreting patterns (themes) within the data.^8^ Following data familiarisation and immersion, a list of codes was generated by S.B. Next, themes were searched for, and an analytical framework was constructed by S.B. Where this framework did not fit the data, themes were further refined and alternative explanations sought until a final framework was agreed by S.B., J.B. and M.L. As well as following standardised topic guides, robust and transparent analysis was critical to ensuring reflexivity and minimising the researcher's influence on emerging themes.

### Ethics

Formal ethical approval was not required as the project formed part of a clinical service evaluation. Approval for this was provided by the senior leadership team at the unit.

## Results

The analytical framework is shown in Table 2 and discussed below. Themes are divided into those that facilitate effective clozapine monitoring, those that act as barriers and those acting as both facilitators and barriers. Pseudonyms are used throughout.

**Table 2:** Analytical framework to emerge from qualitative data collection with staff and patients

Theme	Barrier	Facilitator
Clozapine monitoring as a means of promoting responsibility and independence among patients		✓
Staff are highly motivated to help patients and to promote recovery, despite competing priorities		✓
The roles and responsibilities of different staff groups are not well defined around clozapine monitoring	✓	
Knowledge about clozapine among staff is sometimes lacking	✓	
There is a lack of formal pathways for clozapine physical health monitoring	✓	
Patients have varied understanding and engagement around clozapine	✓	✓

### Facilitators

#### Clozapine monitoring as a means of promoting responsibility and independence among patients

Clozapine care was seen by staff as more than just the therapeutic compound, and about supporting the holistic recovery of an individual. Staff believed that a clozapine clinic would increase patients’ knowledge and emphasise the importance of monitoring. Utilising a clinic arrangement was perceived by staff and stepdown patients as more equivalent to the community, helping patients to adapt upon discharge.

*‘Service users would feel that clozapine was being taken seriously and be reassured by this rather than a random person coming to take their bloods at random time points. It would help them to understand about clozapine’ (Jim, Mental Health Support Worker)*

*‘I think it's a really good idea, it'll be like what happens in the community’ (Matt, stepdown patient)*

Staff believed that patients would respond to the routine of a clinic. Although there might be some pushback initially, it was perceived that it would quickly become the norm.

*‘There may be stumbling blocks at the start, as there is for any new thing, but once it becomes more routine, part of every ward's day, it'll just become normal for everyone’ (Geoff, Mental Health Support Worker)*

#### Staff are highly motivated to help patients and to promote recovery, despite competing priorities

Although all staff groups faced competing pressures on their time and worked in sometimes challenging situations, they mutually perceived each other as well trained, highly competent and motivated. Although ward staff would be required to facilitate clinic attendance, the benefits to patients of an organised system and to the wards of being able to reliably get bloods done were felt to outweigh this. In addition, a clinic was perceived to have benefits for staff development in terms of phlebotomy training, where staff struggled to get supervised experience.

*‘It means the right people will be doing it… it'll be a separate department doing it and will stop the communication problems’ (Lucy, Mental Health Nurse)*

### Barriers

#### The roles and responsibilities of different staff groups are not well defined around clozapine monitoring

All staff groups perceived that the roles and responsibilities of different teams involved in clozapine were not well defined. The exception to this was pharmacy, who were seen to have a clear role in delivering patient education at clozapine initiation and coordinating full blood counts (FBCs) thereafter. No staff group saw themselves as responsible for cardiometabolic monitoring and were not able to identify who was. The main barrier to defining responsibilities was that clozapine monitoring spans mental and physical health. As an antipsychotic, the physical health team saw clozapine as a psychiatric responsibility. By contrast, ward staff believed that it was outside the scope of psychiatry, owing to the physical health monitoring and extensive side-effects.

*‘It's a mental health medication and the responsibility of RMNs. I was always taught that if you're prescribing and administering a medication then it was your responsibility to monitor it’ (Sharon, Physical Health Team)*

Going forward, it *was* believed that any potential clozapine clinic should be staffed by mental and physical health colleagues.

#### Knowledge about clozapine among staff is sometimes lacking

It was perceived by some staff that certain staff groups lacked understanding about areas not viewed to be their responsibility, particularly cardiometabolic monitoring among ward staff. There was concern about this among experienced staff, who believed that undergraduate training around clozapine had declined. They suggested that robust training was needed for forensic staff, given the widespread use of clozapine.

*‘It was drilled in when I was training that you had to ask everyone on clozapine about their bowel habit every morning but I'm not sure they're doing it now’ (Steve, Mental Health Nurse)*

*‘The experienced nurses do this very well. They're well informed about the importance of monitoring clozapine. The new nurses really struggle, they don't know the side effects’ (Kristina, Doctor)*

#### There are a lack of formal pathways for clozapine physical health monitoring

Just as it was not clear who was responsible, it was also believed that pathways for testing and acting upon abnormal physical health results were lacking. Different wards sometimes had different systems, which made it difficult to keep track of how and whether things were done. Staff widely acknowledged that there was a need to organise monitoring, supported by electronic systems. It was believed that it would be difficult to train all staff to do this, and that a clinic model would create a discrete group competent in this.

*‘There's not any formalised process and I think that is probably one of the problems as to why the clinic would be useful I guess’ (Tom, Doctor)*

*‘The same people would be doing it all the time and would know what they were doing’ (Carly, Mental Health Nurse)*

### Both facilitators and barriers

#### Patients have varied understanding and engagement around clozapine

Although most patients were aware that some form of monitoring was required for clozapine, their understanding varied. This was apparent when discussing the reasons for the regular FBCs.

*‘It's for cholesterol isn't it’ (Kyle, low-secure patient)*

*‘It's for the white cell isn't it’ (Warren, low-secure patient)*

*‘It's for the green light isn't it?’ (Elaine, medium-secure patient)*

Aside from regular FBCs, patients had minimal awareness of any other monitoring and were usually only aware of side-effects if they had experienced them.

*‘I can't go to the toilet’ (Colin, medium-secure patient)*

Nevertheless, patients understood why they took clozapine and perceived it favourably for psychiatric symptoms.

*‘Within a few weeks I was more stable and they [hallucinations] disappeared, I sometimes miss them though. Some of them were my mates’ (William, stepdown patients)*

Patients were used to a model of care that required little effort, and staff felt that it could be a struggle to engage some. Likewise, some patients described the convenience of the current system.

*‘We struggle to get patients to go and see the GP. It's a challenge just to get people out of bed and to come to the ward clinic room’ (Steve, Mental Health Nurse)*

*‘I'm quite happy with how it is at the moment… more convenient’ (Kyle, low-secure patient)*

## Discussion

**Fig. 1:**
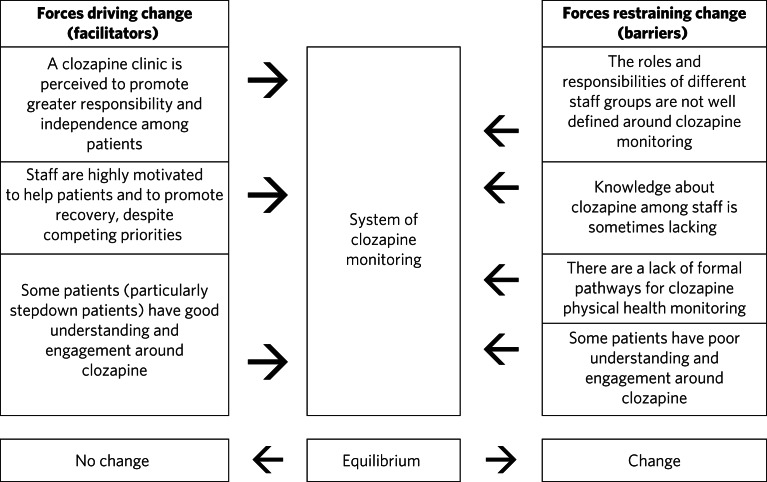
Force field analysis of forces driving and resisting change in relation to clozapine monitoring.^12,13^

Effective monitoring of physical health parameters and side-effects is a must-do aspect of clozapine care to prevent serious incidents in the short term, as well as the long-term health effects of cardiometabolic complications. Despite this, studies repeatedly report that adherence to the required monitoring, in both in-patient and community settings, is incomplete.^2–6,10,11^ Although clozapine is widely used in forensic settings, there is very little published literature relating to its use here. This qualitative study has provided valuable insight into clozapine monitoring in a secure unit and has identified facilitators and barriers to effective monitoring. These are displayed in Fig. 1 in the format of Lewin's force field analysis, which depicts change as a state of imbalance between driving and resisting forces, with change achieved by increasing the facilitators, reducing the barriers, or both.^12,13^

The key facilitator was the motivation of staff to facilitate recovery and prepare patients for discharge. Staff understood the risks of not effectively monitoring clozapine and were keen to implement a more efficient model. This mirrors the findings of a previous evaluation of community shared-care clozapine monitoring, where forensic healthcare professionals were motivated by enabling patients to develop skills for independence.^7^ Both staff and patients believed that physical and mental healthcare were equally important for people taking clozapine.^7^ In our study, there was a range of understanding among patients, with stepdown patients having greater understanding of the associated benefits of monitoring. Their engagement is a further key facilitator to be harnessed. Linked to this, staff noted that patients responded favourably to routine, and that consistency should be a core component of clozapine monitoring.

In terms of barriers, an important finding was that roles and responsibilities for monitoring were not clearly defined and inter-team communication was sometimes lacking. Spanning mental and physical health, clozapine monitoring was widely perceived to be outside the scope of practice of the different teams involved. These factors have previously been identified as key determinants of psychiatrists’ practice in relation to clozapine, and major contributors to low rates and inconsistency of follow-up.^11^ Likewise, there was sometimes a lack of knowledge about clozapine monitoring, particularly aspects that staff did not perceive as their responsibility. Although the FBC component was widely understood, as results must be available to enable clozapine dispensing, some staff perceived understanding of cardiometabolic monitoring to be limited among ward staff, despite the widespread prevalence of these complications. Defined roles and responsibilities must be supported by robust pathways for clozapine monitoring. Inconsistent documentation, limited knowledge about clozapine and a lack of communication between teams have previously been shown to limit improvements when abnormalities are detected.^4,6^ Logistically, access to phlebotomy-trained staff was a key barrier to on-schedule monitoring, which has been highlighted in previous audits of clozapine monitoring.^2,3^ From a patient perspective, monitoring was passive, and many had little insight into the monitoring requirements. Although there were more barriers than facilitators with respect to changing the clozapine monitoring system, the facilitators were strong motivators, so it is anticipated that they will drive the proposed change and allow the barriers identified to be overcome.

Although not widely explored in the academic literature, there is some consensus as to what gold-standard clozapine monitoring encompasses, and this study adds to this. First, it is essential that staff and patients perceive clozapine monitoring as a tool for facilitating patient recovery, independence and safety, and not simply as the process for supplying a medication.^7^ Patient education should not be a one-off event but should be repeated throughout treatment, especially as patients may be unwell at the time of clozapine initiation and have low health literacy.^14^ Clozapine monitoring must be supported by care pathways and effective interventions to ensure that, first, monitoring takes place and, second, that abnormal findings are actioned.^6^ Pathways should be standardised so that they can be easily followed by busy staff working across wards. Any system of monitoring must be supported by electronic tools to keep track of monitoring.^6^ An in-patient clozapine clinic is an effective system for clozapine monitoring that is widely supported by staff and patients. This provides FBC monitoring, along with monitoring of other physical health parameters and side-effects. In terms of staffing, multidisciplinary representation is likely to be effective, with mental and physical health co-staffing supported in this study. Several senior staff members in our study raised concerns about undergraduate training around clozapine. Given the complex monitoring and severe side-effects, robust training on clozapine should be encompassed by undergraduate mental health nursing degrees and a mandatory workplace training module. Where establishment of a clozapine clinic is not feasible, it is recommended that, as a minimum, a clozapine pathway is established that defines the roles and responsibilities of different staff groups and triggers appropriate communication and actions where abnormal results are identified. This should be underpinned by an electronic system that simplifies the process and is accessible by the relevant staff.

### Limitations

As with much qualitative work, there were small numbers of participants, and they were interviewed in a single location. This restricts the transferability of results, as some may be specific to the individual setting. However, the findings reinforce those from the wider literature, and beliefs and challenges have been identified that are widely applicable to mental health settings.

A further limitation is the possibility that the results were influenced by recall and social desirability bias. Furthermore, as patients were approached by a senior nurse, it is possible that patients with a favourable attitude towards clozapine or a good relationship with staff were recruited.

### Summary

Clozapine is widely used in forensic settings, yet considerable challenges remain around its use, particularly ensuring on-schedule monitoring of physical health parameters. The reasons underlying this have not previously been widely explored, and this qualitative study adds to the evidence base by identifying facilitators of and barriers to monitoring. Forensic healthcare staff are highly motivated to promote skills for independence and recovery among patients, and understand the role of clozapine care in this. This motivation and enthusiasm is a core facilitator of positive change and increases the likelihood that change will be sustained. Formal procedures and pathways must be in place to underlie clozapine monitoring, supported by electronic systems and tools. A clozapine clinic is a robust mechanism for providing in-patient clozapine monitoring in secure settings that is widely supported by staff and patients, and prepares patients for transition to the community. These findings can be applied to other mental health units to optimise their systems of clozapine monitoring.

## Data Availability

The data that support the findings of this study are available upon reasonable request from the corresponding author, S.B. The data are not publicly available due to their containing information that could compromise the privacy of participants.

## About the authors

**Sarah Blagden** is Public Health Specialty Registrar at Health Education North West, Liverpool, UK, and Lancashire and South Cumbria NHS Foundation Trust, Preston, UK. **Jane Beenstock** is Consultant in Public Health at Lancashire and South Cumbria NHS Foundation Trust, Preston; and the Division of Health Research, Lancaster University, Lancaster, UK. **Natalie Auld** is Pharmacy Technician at Lancashire and South Cumbria NHS Foundation Trust, Preston, UK. **Steve Noblett** is Consultant Forensic Psychiatrist at Lancashire and South Cumbria NHS Foundation Trust, Preston, UK. **Mark Limmer** is Senior Lecturer in Public Health at the Division of Health Research, Lancaster University, Lancaster, UK.

## Appendices

### Appendix 1 Staff topic guide (interviews and focus groups)

**Clozapine monitoring – staff topic guide (interviews and focus groups)**

- Can you tell me about your role?
- Can you tell what you understand about clozapine monitoring?
  - Prompts:
    - Why do you think clozapine monitoring is important?
    - What are the risks to patients if monitoring is not done correctly?
- Can you tell me about your role with regards to clozapine?
- What is your understanding of how clozapine is currently monitored here?
  - Prompts:
    - Full blood counts
    - Physical health parameters, side-effects
    - Patient education
- Do you think the current system of monitoring works well?
- Who do you think should be responsible for clozapine monitoring here?
  - Prompts:
    - Full blood counts
    - Physical health parameters
    - Side-effects
    - What do you think is the role of the ward staff, physical health team, pharmacy, consultant and medical staff?
- What do you think is the role of the service user in monitoring their clozapine?
  - Prompts:
    - Do many patients self-medicate?
    - How are patients on clozapine prepared for discharge?
    - Do you think service users should be more involved in monitoring their clozapine?
    - Do you think service users are given sufficient education about clozapine when they are initiated on it and throughout their treatment?
- What do you understand about how clozapine is electronically monitored?
  - Prompts:
    - FBCs
    - Physical health bloods, clozapine levels, ECGs etc
    - Side-effects
    - How do you think this could be improved?
- Are there any aspects of clozapine monitoring that you think work particularly well here?
- Are there any aspects of clozapine monitoring that you think would benefit from improvement?
- What barriers do you think there are to clozapine monitoring here?
- Can you think of anything that could be done to improve the system of clozapine monitoring here?
- What do you think about the idea of a clozapine clinic that service users attend for all aspects of clozapine monitoring?
  - Prompts:
    - Where do you think this should be?
    - Who do you think should run this?
    - Would this work for all service users?
- Is there anything else with regards to clozapine that we haven't discussed and that you would like to mention?

### Appendix 2 Service user interview topic guide

- Can you tell me what you understand about clozapine?
  - Prompts:
    - Why do you think you take clozapine?
    - How long have you been taking clozapine for?
    - Has clozapine worked well for your symptoms?
- Did you start clozapine during this admission or previously?
- When you started on clozapine, what information was given to you about it?
  - Prompts:
    - Who gave you this information?
    - Was this spoken or written information?
- Can you tell me what you understand about the side-effects of clozapine?
  - Prompts:
    - Were you given information about side-effects before you started taking clozapine?
    - Were you given information about how to prevent any side-effects?
- Have you experienced any side-effects from taking clozapine?
  - Tell me about this
- Can you tell me about the monitoring that you have to have for clozapine?
  - Prompts:
    - How often does this happen?
    - Do you understand what they're monitoring for?
    - Do you receive the results of your blood tests?
    - Who do you think is responsible for monitoring your clozapine?
- Aside from the regular blood tests for the green, amber, red result, do you have any other monitoring for clozapine?
  - Prompts:
    - Do staff on the ward measure your weight and ask about your bowels?
    - Do you have blood tests for other things as part of your clozapine monitoring? (blood sugar, cholesterol etc)
- Low secure and step-down only – can you tell me what you understand about how your clozapine will be monitored and dispensed in the community when you leave hospital?
- What do you think about the idea of having a clinic on the hospital site where you'd go to have your clozapine monitoring done?
- Is there anything else about clozapine that we haven't mentioned and that you'd like to discuss?
